# 4-Amino-3,5-di­chloro­pyridine

**DOI:** 10.1107/S2414314624011209

**Published:** 2024-11-22

**Authors:** Thankappan Ramalakshmi Anantheeswary, Sundaramoorthy Gomathi, Ramu Shyamaladevi, Samson Jegan Jennifer, Ibrahim Abdul Razak

**Affiliations:** a Department of Chemistry, Periyar Maniammai Institute of Science & Technology, Thanjavur, Tamilnadu-613403, India; b Department of Chemistry, Bishop Heber College, Tiruchirappalli, Tamilnadu-620017, India; c X-ray Crystallography Unit School of Physics University Sains Malaysia 11800 USM Penang Malaysia; Howard University, USA

**Keywords:** 4-amino-3,5-di­chloro­pyridine, crystal structure, offset π–π stacking, halogen–π inter­action. Hirshfeld surface analysis

## Abstract

The title compound, C_5_H_4_Cl_2_N_2_, crystallizes with one mol­ecule in the asymmetric unit. In the crystal, the mol­ecular entities are assembled through strong N—H⋯N hydrogen bonding, forming supra­molecular chains extending along the *b-*axis direction. These chains are inter­connected by offset π–π stacking inter­actions and consolidated by halogen–π inter­actions. The mol­ecular inter­actions were qu­anti­fied by Hirshfeld surface analysis, showing the significant contributions of Cl⋯H/H⋯Cl (40.1%), H⋯H (15.7%) and N⋯H / H⋯N (13.1%) inter­actions. Energy framework analysis using the CE-B3LYP/6–31 G(d,p) basis set revealed that Coulombic inter­actions make a considerable contribution to the total energy and crystal packing.

## Abstract 2

The crystal structure of 4-amino-3,5-di­chloro­pyridine was determined by single-crystal X-ray diffraction technique and its mol­ecular inter­actions were investigated.

## Graphical abstract

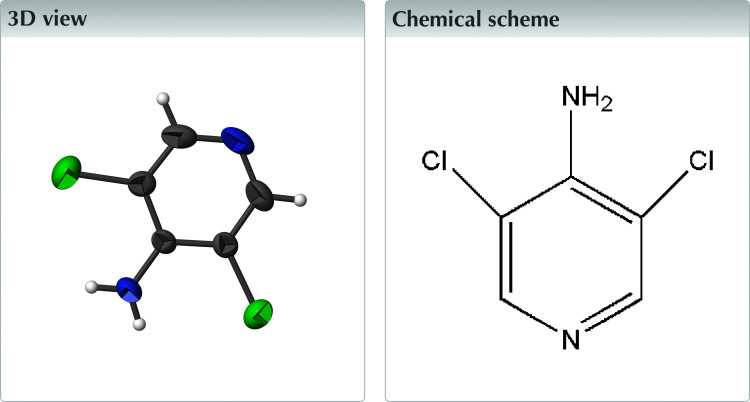

## Structure description

4-Amino-3,5-di­chloro­pyridine (ADCP) is of inter­est in organic synthesis and medicinal chemistry due to its versatile reactivity and potential properties including anti­microbial (Singaram *et al.*, 2016 ▸) and anti-cancer (Onnis *et al.*, 2009 ▸) activity. Its derivatives are employed in the development of drugs targeting various biological inflammatory diseases (Boland *et al.*, 2014 ▸), bacterial infections (Chung *et al.*, 1999 ▸) and hyperthyroidism. Structural studies of chloro and di­chloro­pyridine derivatives, namely 2-amino-3-chloro­pyridine (Hu *et al.*, 2011 ▸), 2-amino-5-chloro­pyridine-fumaric acid (Hemamalini & Fun, 2010 ▸), 2-amino-3,5-di­chloro­pyridinium chloride monohydrate (Anagnostis & Turnbull, 1998 ▸) and 4-amino-3,5-di­chloro­pyridinium 3-hy­droxy­pico­linate monohydrate (Ashokan *et al.*, 2023 ▸) have been reported in the literature. The crystal structure of ADCP will be helpful in identifying the structural and supra­molecular patterns that play a significant role in its functional properties.

**Figure 1:**
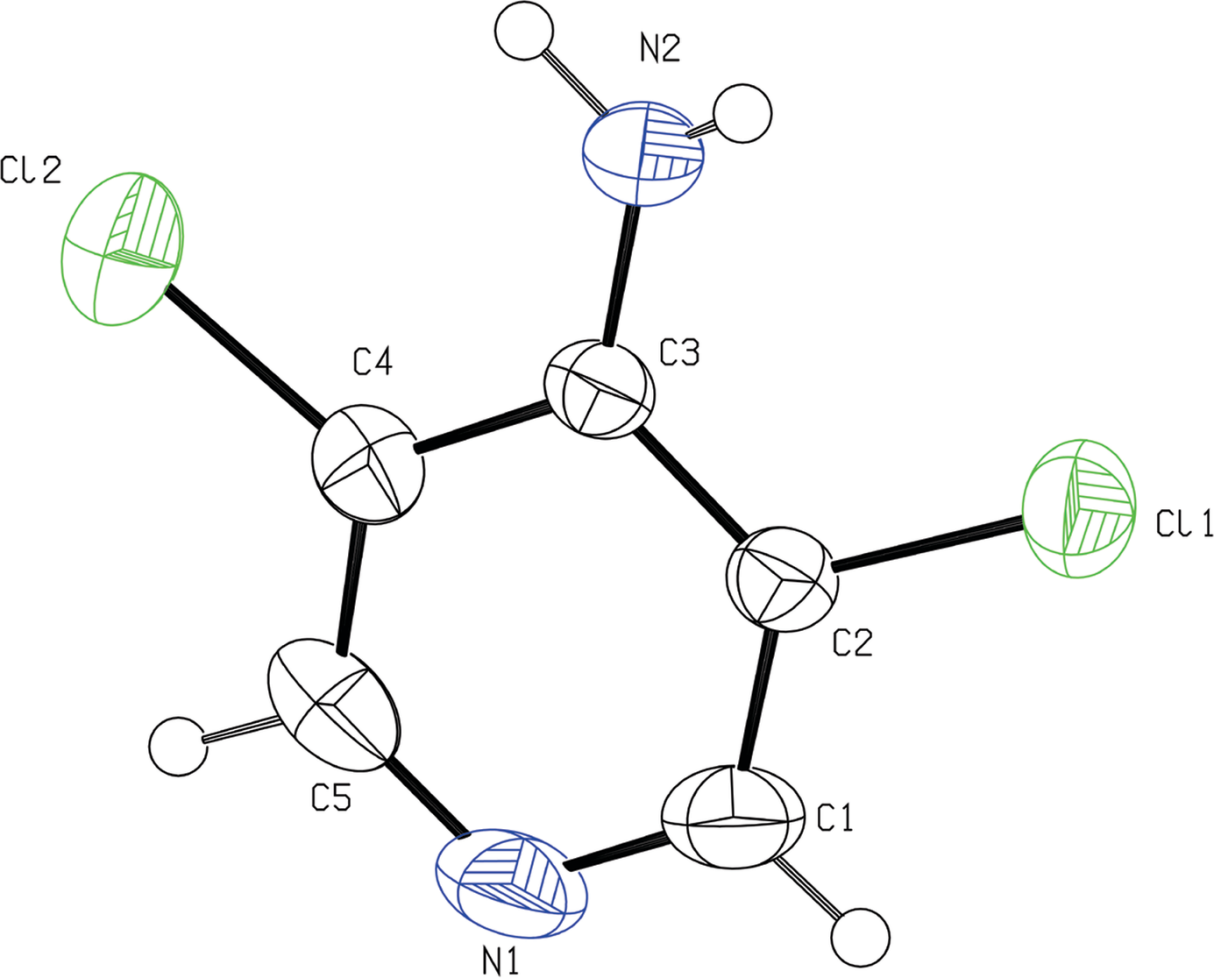
*ORTEP* view of ADCP with displacement ellipsoids drawn at the 50% probability level.

**Figure 2:**
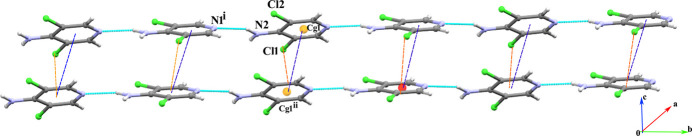
Supra­molecular chains formed through N—H⋯N hydrogen bonds and inter­linked *via* offset aromatic π–π stacking and halogen–π inter­actions. [Symmetry codes: (i) 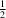 − *x*, 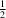 + *y*, 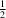 + *z*; (ii) *x*, *y*, 1 + *z*.]

The asymmetric unit of ADCP (Fig. 1 ▸) consists of one mol­ecule. The C1—N1—C5 bond angle is 116.4 (5)°, indicating that the ring nitro­gen atom of ADCP is *sp*^2^ hybridized (Newell *et al.*, 2022 ▸). The deviation from the ideal angle of 120° is due to the strain exerted by the presence of a lone pair on the nitro­gen atom and contributes to the weak basicity of ADCP. In the crystal, the mol­ecular entities are assembled through N—H⋯N hydrogen bonding between the amino N2 atom and ring N1 atom of a symmetry-related mol­ecule (Table 1 ▸), forming supra­molecular chains along the *b-*axis direction. Neighbouring chains are inter­linked by offset aromatic π–π stacking inter­actions (Malenov & Zarić, 2023 ▸) between the pyridine π clouds [*Cg*1⋯*Cg*1(*x*, *y*, 1 + *z*) = 3.8638 (19) Å, perpendicular distance = 3.4954 (12) Å and slip angle = 25.2° where *Cg*1 is the centroid of the N1/C1–C5 ring; symmetry code: *x*, *y*, 1 + *z*], as shown in Fig. 2 ▸. The cohesion of the crystal structure is enhanced by the presence of halogen–π inter­actions (Rahman *et al.*, 2003 ▸) with a Cl⋯π distance of = 3.9375 (17) Å.

**Figure 3:**
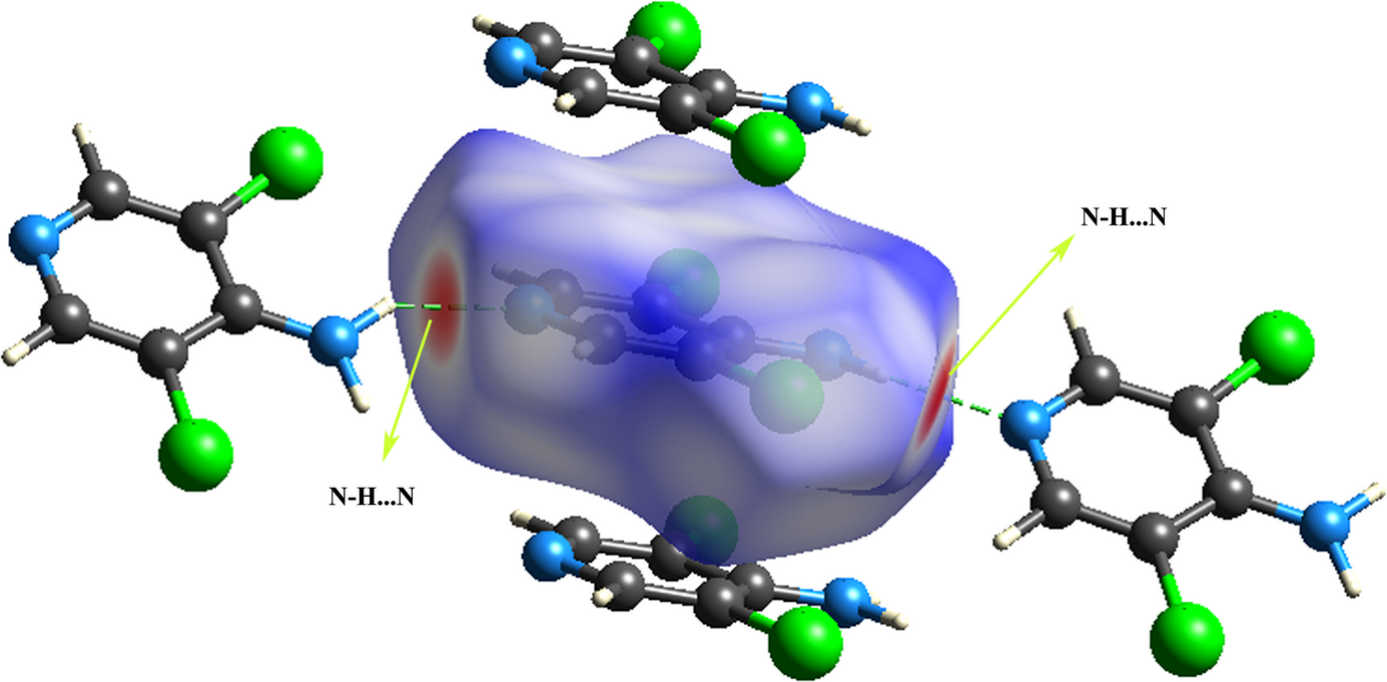
Hirshfeld surface mapped over *d*_norm_ for ADCP.

Hirshfeld surface (HS) (McKinnon *et al.*, 2007 ▸; Spackman & Jayatilaka, 2009 ▸) analysis was used to visualize and qu­antify the inter­molecular inter­actions in the crystal. The isovalue of w(*r*)= 1/2, mapped over *d*_norm_ on the HS to the inside (*d*_i_) and exterior (*d*_e_) atoms between the arbitrary units (−0.46 to 1.21) specifies the HS of ADCP. The intensity of the hydrogen-bonded contacts in the HS of ADCP are represented as red, blue, and white in Fig. 3 ▸. The strongest inter­actions are indicated by red, weaker inter­actions are represented by white, and inter­actions larger than the total of the van der Waals radii of neighbouring atoms are indicated by blue. The acceptor regions of hydrogen bonds are highlighted in red on the *d*_norm_ surface. The HS plotted over *d*_norm_ shows the atoms within 3.8 Å of the HS, showing the strong N—H⋯N inter­molecular hydrogen-bonding inter­actions linking 4-amino N2 and ring N1 atoms of adjacent ADCP mol­ecules.

**Figure 4:**
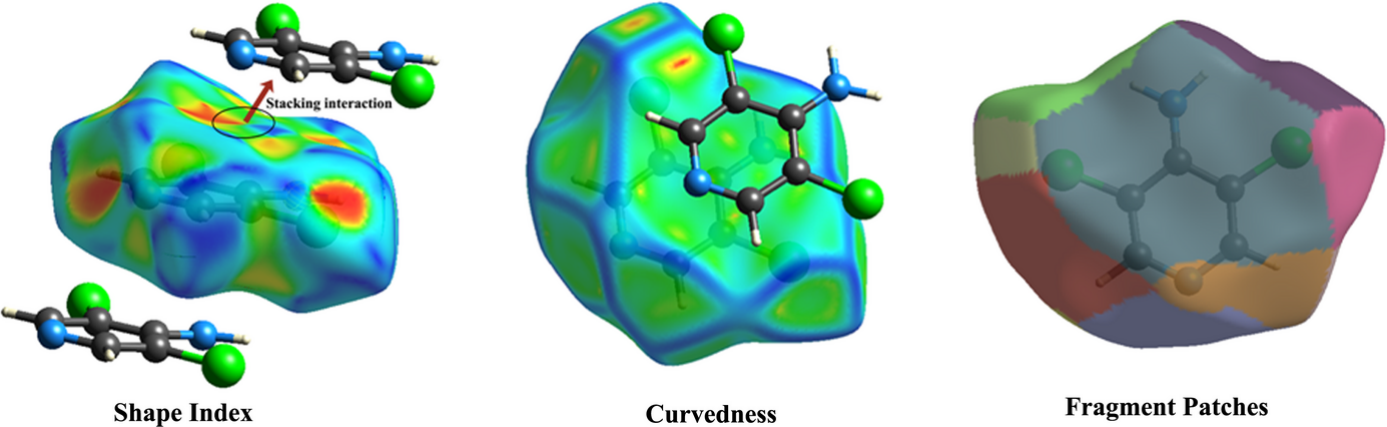
Shape-index, curvedness and colour patches of the mol­ecule within 3.8. Å.

The presence of π–π stacking inter­actions is indicated and confirmed by the contiguous red and blue triangular regions around the pyridine rings on the HS mapped over the shape index (see Fig. 4 ▸). The stacking inter­action is also supported by the large, flat green regions around the pyridine ring on the corresponding curvedness surface. Colour patches on the Hirshfeld surface depend on their closeness to the adjacent mol­ecules and provide information regarding the nearest coordination environment of a mol­ecule. The atoms within 3.8 Å from the HS of ADCP with their respective mol­ecules, involving non-covalent inter­actions at various levels are displayed in different colour codes in Fig. 4 ▸.

**Figure 5:**
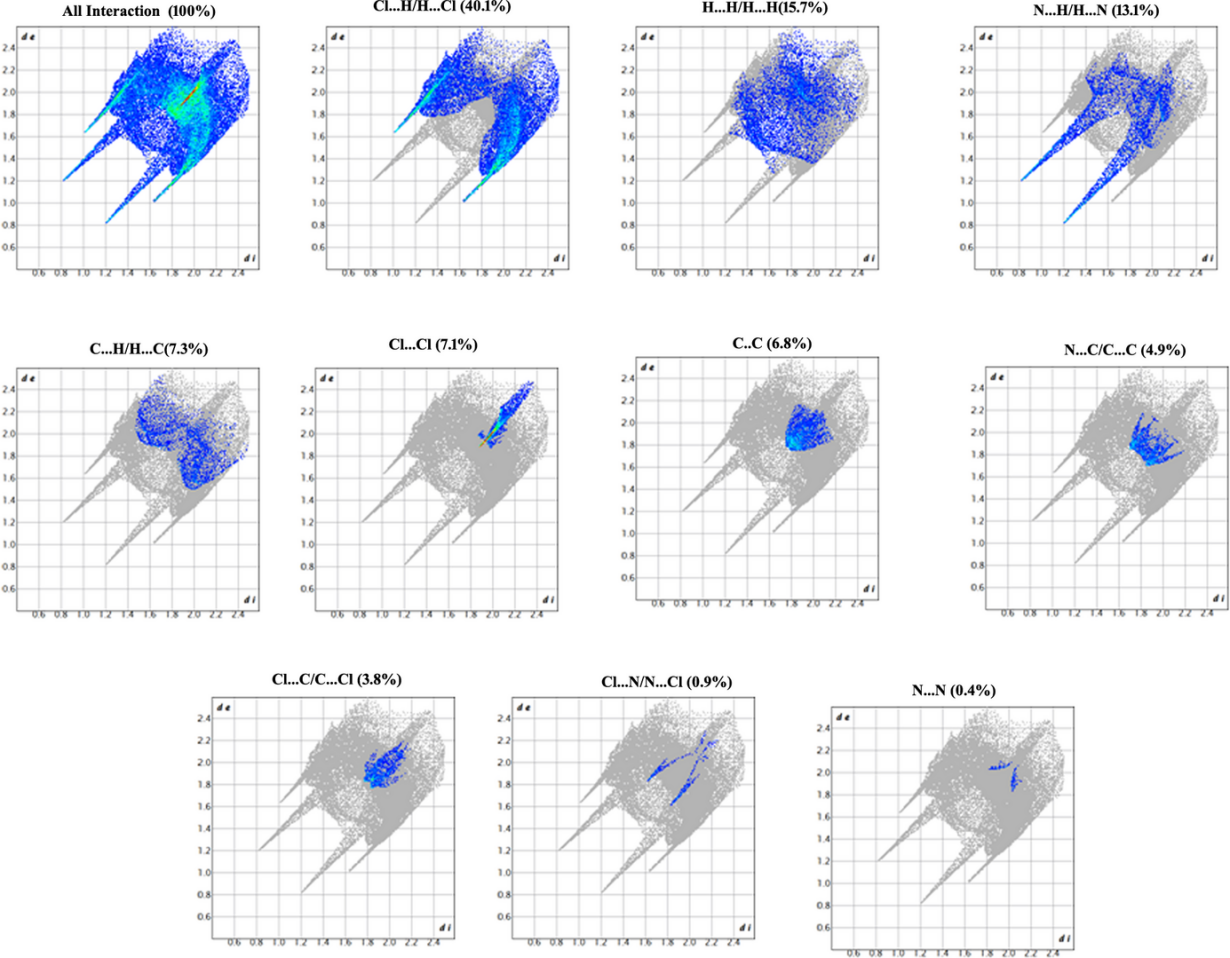
Two-dimensional fingerprint plots of the mol­ecule with percentage contributions.

The total inter­molecular inter­actions between the promolecule and the exterior mol­ecules were qu­anti­fied by two-dimensional fingerprint plots (Spackman & McKinnon, 2002 ▸) in terms *d*_i_ and *d*_e_ and are represented as blue regions with dots of varied intensities in Fig. 5 ▸. In the fingerprint plots, among all the non-covalent inter­actions, Cl⋯H/H⋯Cl (40.1%) followed by H⋯H (15.7%) contacts contribute the maximum in the crystal packing of ADCP. The N⋯H/H⋯N contacts provide a significant contribution (13.1%) through the strong hydrogen bonding involving N and H atoms. The C⋯H / H⋯C (7.3%), Cl⋯Cl (7.1%),C⋯C (6.8%), N⋯C/C⋯N (4.9%) and Cl⋯C/C⋯Cl (3.8%) inter­actions also contribute to the cohesion of the crystal structure.

The stability of the crystal packing arrangement is achieved by the systematic balance between the mol­ecules by which the mol­ecules are aligned to increase the attractive inter­actions and decrease the repulsive forces to yield the stable and energetically favoured crystalline structure. The total inter­action energy (kJ mol^−1^) is the sum of electrostatic energy, polarization energy, dispersion energy and repulsion energy. The total energy was calculated using the CE-B3LYP/6–31 G(d,p) basis set implemented in *Crystal Explorer 17.5* (Turner *et al.*, 2017 ▸) by computing the individual components using the monomer wavefunctions that have been appropriately scaled (*k*_ele_= 1.057, *k*_pol_= 0.740, *k*_dis_= 0.871 and *k*_rep_= 0.618) to reproduce the counterpoise-corrected energies B3LYP/6–31 G(d,p) with a small mean absolute deviation of 2.4 kJ mol^−1^ (Mackenzie *et al.*, 2017 ▸). The various colour codes in Table 2 ▸ indicate the exterior inter­acting mol­ecules around the distance between mol­ecular centroids (*R*) and the summation of the scaled values of the individual energy components of exterior mol­ecule is given as *E*_tot._

**Figure 6:**
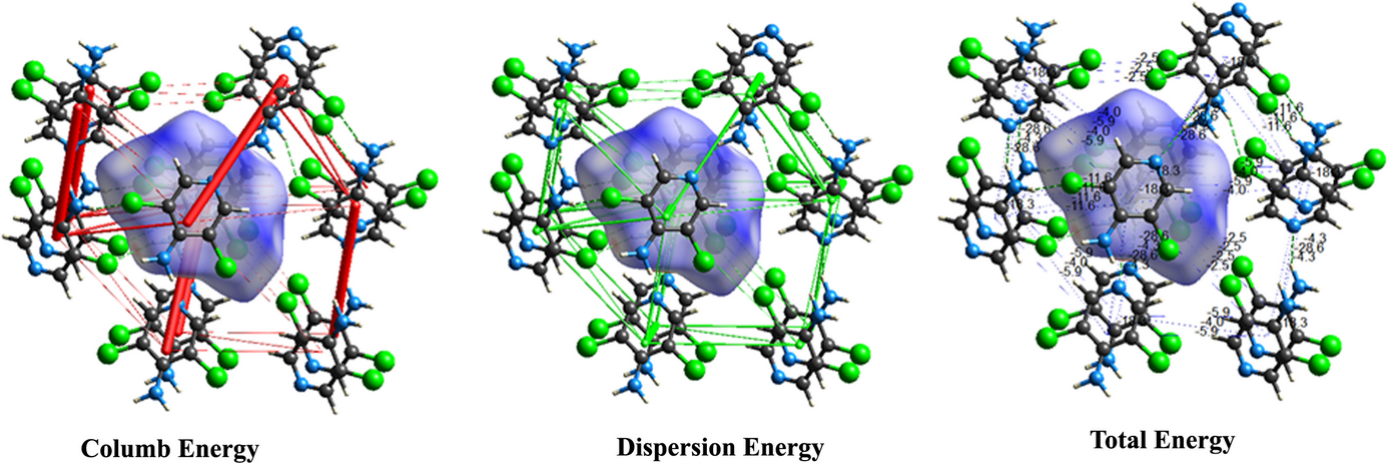
Energy frameworks for ADCP.

The energy framework analysis reveals the strength of the inter­molecular inter­actions contributing to the crystal packing of the promolecule and its 13 exterior inter­acting mol­ecules, forming a mol­ecular assembly of 14 mol­ecules. The total and individual energy components of these mol­ecular assemblies were calculated using CE-B3LYP/6–31 G(d,p), resulting in energy frameworks for Coulombic energy, dispersion energy and total energy. These are represented by scaled cylinders with a reference scale of 100. The dimensions of these cylinders reflect the magnitude of the vectorial inter­action energy. Fig. 6 ▸ shows that electrostatic (Coulombic) inter­actions make a more significant contribution to the total energy and crystal packing than dispersion inter­actions among neighbouring mol­ecules.

## Synthesis and crystallization

4-Amino-3,5-di­chloro­pyridine (0.04075 mg) was dissolved in 20 ml of water and warmed over a water bath for 20 min at 353 K. The solution was then allowed to cool slowly at room temperature. After a few days, colourless crystals were separated out from the mother liquor.

## Refinement

Crystal data, data collection and structure refinement details are summarized in Table 3 ▸.

## Supplementary Material

Crystal structure: contains datablock(s) global, I. DOI: 10.1107/S2414314624011209/bv4053sup1.cif

Structure factors: contains datablock(s) I. DOI: 10.1107/S2414314624011209/bv4053Isup2.hkl

Supporting information file. DOI: 10.1107/S2414314624011209/bv4053Isup3.cml

CCDC reference: 2403603

Additional supporting information:  crystallographic information; 3D view; checkCIF report

## Figures and Tables

**Table 1:** Hydrogen-bond geometry (Å, °)

*D*—H⋯*A*	*D*—H	H⋯*A*	*D*⋯*A*	*D*—H⋯*A*
N2—H1*A*⋯Cl1^i^	0.84 (4)	2.81 (4)	3.625 (3)	164 (3)
N2—H2*A*⋯N1^ii^	0.85 (2)	2.16 (3)	2.931 (3)	149 (3)

Symmetry codes: (i) 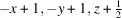; (ii) 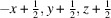.

**Table 2:** The inter­action energies (kJ mol^−1^) of the promolecule with the surrounding mol­ecules within 3.8 Å *N* = number of pairs with that energy; symmetry operation relates that particular colour-coded mol­ecule with the central mol­ecule; *R* is the distance (in Å) between mol­ecular centroids (mean atomic position). B3LYP/6–31G(d,p) electron density was used with scale factors 1.057 (*k*_ele_), 0.740 (*k*_pol_), 0.871 (*k*_dis_) and 0.618 (*k*_rep_).

Colour	*N*	Symmetry operation	*R*	*E*_ele_	*E*_pol_	*E*_dis_	*E*_rep_	*E*_tot_
Red	2	*x* + 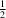 , −*y* + 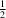 , *z*	8.30	−2.4	−0.1	−4.4	3.9	−4.0
Yellow	2	-*x*, −*y*, *z* + 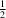	8.88	−1.2	−0.1	−2.9	2.2	−2.5
Fluoro­green	2	*x* + 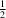 , −*y* + 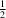 , *z*	7.34	−2.2	−0.1	−8.1	5.8	−5.9
Green	2	-*x* + 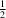 , *y* + 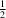 , *z* + 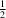	6.87	−33.8	−8.3	−11.4	37.6	−28.6
Blue	2	-*x* + 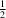 , *y* + 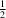 , *z* + 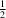	6.87	−0.4	−0.4	−5.7	2.1	−4.3
Dark blue	2	*x*, *y*, *z*	3.86	−0.7	−0.8	−33.5	19.6	−18.3
Pink	2	-*x*, −*y*, *z* + 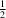	6.57	−8.7	−1.5	−10.7	12.9	−11.6

**Table 3:** Experimental details

Crystal data	
Chemical formula	C_5_H_4_Cl_2_N_2_
*M*_r_	163.00
Crystal system, space group	Orthorhombic, *Pna*2_1_
Temperature (K)	296
*a*, *b*, *c* (Å)	13.304 (2), 12.911 (2), 3.8636 (7)
*V* (Å^3^)	663.64 (19)
*Z*	4
Radiation type	Mo *K*α
μ (mm^−1^)	0.88
Crystal size (mm)	0.33 × 0.23 × 0.22

Data collection	
Diffractometer	Bruker APEXII CCD
No. of measured, independent and observed [*I* > 2σ(*I*)] reflections	5840, 1969, 1729
*R*_int_	0.017
(sin θ/λ)_max_ (Å^−1^)	0.709

Refinement	
*R*[*F*^2^ > 2σ(*F*^2^)], *wR*(*F*^2^), *S*	0.031, 0.116, 0.88
No. of reflections	1969
No. of parameters	90
No. of restraints	4
H-atom treatment	H atoms treated by a mixture of independent and constrained refinement
Δρ_max_, Δρ_min_ (e Å^−3^)	0.26, −0.22
Absolute structure	Flack (1983 ▸)
Absolute structure parameter	−0.02 (3)

Computer programs: *APEX2* and *SAINT* (Bruker, 2016 ▸), *SHELXT* (Sheldrick, 2015*a* ▸), *SHELXL* (Sheldrick, 2015*b* ▸), *PLATON* (Spek, 2020 ▸), *Mercury* (Macrae *et al.*, 2020 ▸), *POVRay* (Cason, 2004 ▸) and *publCIF* (Westrip, 2010 ▸).

## full crystallographic data

### 3,5-Dichloropyridin-4-amine Crystal data

**Table:**

C_5_H_4_Cl_2_N_2_	*F*(000) = 328
*M**_r_* = 163.00	*D*_x_ = 1.631 Mg m^−^^3^
Orthorhombic, *P**n**a*2_1_	Mo *K*α radiation, λ = 0.71073 Å
Hall symbol: P 2c -2n	Cell parameters from 1969 reflections
*a* = 13.304 (2) Å	θ = 2.2–30.3°
*b* = 12.911 (2) Å	µ = 0.88 mm^−^^1^
*c* = 3.8636 (7) Å	*T* = 296 K
*V* = 663.64 (19) Å^3^	Block, colourless
*Z* = 4	0.33 × 0.23 × 0.22 mm

### 3,5-Dichloropyridin-4-amine Data collection

**Table:**

Bruker APEXII CCD diffractometer	*R*_int_ = 0.017
φ and ω scans	θ_max_ = 30.3°, θ_min_ = 2.2°
5840 measured reflections	*h* = −17→18
1969 independent reflections	*k* = −16→18
1729 reflections with *I* > 2σ(*I*)	*l* = −5→5

### 3,5-Dichloropyridin-4-amine Refinement

**Table:**

Refinement on *F*^2^	Hydrogen site location: mixed
Least-squares matrix: full	H atoms treated by a mixture of independent and constrained refinement
*R*[*F*^2^ > 2σ(*F*^2^)] = 0.031	W = 1/[Σ^2^(*F*O^2^) + (0.1*P*)^2^] WHERE *P* = (*F*O^2^ + 2*F*C^2^)/3
*wR*(*F*^2^) = 0.116	(Δ/σ)_max_ = 0.001
*S* = 0.88	Δρ_max_ = 0.26 e Å^−^^3^
1969 reflections	Δρ_min_ = −0.22 e Å^−^^3^
90 parameters	Absolute structure: Flack (1983)
4 restraints	Absolute structure parameter: −0.02 (3)

### 3,5-Dichloropyridin-4-amine Special details

**Table:**

Geometry. Bond distances, angles etc. have been calculated using the rounded fractional coordinates. All su's are estimated from the variances of the (full) variance-covariance matrix. The cell esds are taken into account in the estimation of distances, angles and torsion angles
Refinement. Refinement on F^2^ for ALL reflections except those flagged by the user for potential systematic errors. Weighted R-factors wR and all goodnesses of fit S are based on F^2^, conventional R-factors R are based on F, with F set to zero for negative F^2^. The observed criterion of F^2^ > 2sigma(F^2^) is used only for calculating -R-factor-obs etc. and is not relevant to the choice of reflections for refinement. R-factors based on F^2^ are statistically about twice as large as those based on F, and R-factors based on ALL data will be even larger.

### 3,5-Dichloropyridin-4-amine Fractional atomic coordinates and isotropic or equivalent isotropic displacement parameters (Å^2^)

**Table:**

	*x*	*y*	*z*	*U*_iso_*/*U*_eq_	
Cl1	0.49802 (4)	0.35972 (5)	0.7165 (4)	0.0500 (2)	
Cl2	0.11701 (5)	0.46637 (7)	0.3776 (3)	0.0665 (3)	
N1	0.2719 (2)	0.20589 (16)	0.2911 (8)	0.0568 (8)	
N2	0.32466 (18)	0.50542 (15)	0.6648 (8)	0.0466 (7)	
C1	0.3604 (2)	0.23315 (19)	0.4280 (10)	0.0508 (8)	
C2	0.37987 (16)	0.33067 (18)	0.5504 (7)	0.0368 (6)	
C3	0.30703 (16)	0.40870 (15)	0.5454 (6)	0.0337 (6)	
C4	0.21489 (18)	0.37787 (18)	0.4008 (7)	0.0398 (6)	
C5	0.2015 (2)	0.2790 (2)	0.2801 (8)	0.0516 (8)	
H1	0.41105	0.18363	0.44048	0.0610*	
H1A	0.373 (2)	0.525 (3)	0.788 (12)	0.081 (16)*	
H2A	0.2797 (17)	0.5525 (19)	0.653 (10)	0.060 (11)*	
H5	0.13951	0.26201	0.18478	0.0620*	

### 3,5-Dichloropyridin-4-amine Atomic displacement parameters (Å^2^)

**Table:**

	*U*^11^	*U*^22^	*U*^33^	*U*^12^	*U*^13^	*U*^23^
Cl1	0.0377 (3)	0.0565 (4)	0.0559 (4)	0.0016 (2)	−0.0031 (3)	0.0049 (3)
Cl2	0.0437 (4)	0.0687 (5)	0.0870 (6)	0.0093 (3)	−0.0093 (4)	0.0003 (5)
N1	0.0671 (14)	0.0350 (9)	0.0683 (17)	−0.0125 (10)	0.0056 (14)	−0.0128 (10)
N2	0.0431 (12)	0.0317 (9)	0.0651 (16)	−0.0004 (8)	−0.0017 (11)	−0.0128 (10)
C1	0.0611 (16)	0.0315 (10)	0.0598 (16)	−0.0001 (10)	0.0102 (15)	−0.0035 (11)
C2	0.0369 (11)	0.0323 (9)	0.0412 (11)	−0.0019 (8)	0.0045 (10)	0.0006 (9)
C3	0.0357 (10)	0.0287 (9)	0.0366 (10)	−0.0035 (8)	0.0047 (9)	−0.0012 (8)
C4	0.0372 (11)	0.0420 (10)	0.0401 (12)	−0.0045 (8)	0.0016 (10)	−0.0007 (10)
C5	0.0512 (14)	0.0517 (13)	0.0518 (15)	−0.0208 (12)	0.0009 (12)	−0.0060 (12)

### 3,5-Dichloropyridin-4-amine Geometric parameters (Å, º)

**Table:**

Cl1—C2	1.739 (2)	N2—H2A	0.85 (2)
Cl2—C4	1.735 (3)	N2—H1A	0.84 (4)
N1—C1	1.338 (4)	C3—C4	1.405 (3)
N1—C5	1.330 (4)	C4—C5	1.371 (4)
N2—C3	1.352 (3)	C1—H1	0.9300
C1—C2	1.370 (4)	C5—H5	0.9300
C2—C3	1.398 (3)		
			
C1—N1—C5	116.5 (2)	N2—C3—C4	123.3 (2)
N1—C1—C2	123.0 (2)	Cl2—C4—C3	119.27 (17)
Cl1—C2—C1	119.78 (18)	Cl2—C4—C5	119.9 (2)
Cl1—C2—C3	118.44 (17)	C3—C4—C5	120.8 (2)
C1—C2—C3	121.8 (2)	N1—C5—C4	124.0 (3)
C3—N2—H1A	127 (3)	N1—C1—H1	119.00
C3—N2—H2A	121.2 (18)	C2—C1—H1	118.00
H1A—N2—H2A	111 (3)	N1—C5—H5	118.00
C2—C3—C4	113.97 (19)	C4—C5—H5	118.00
N2—C3—C2	122.7 (2)		
			
C5—N1—C1—C2	−0.4 (5)	C1—C2—C3—C4	−0.9 (4)
C1—N1—C5—C4	−0.4 (5)	N2—C3—C4—Cl2	−1.1 (4)
N1—C1—C2—Cl1	−178.8 (3)	N2—C3—C4—C5	179.3 (3)
N1—C1—C2—C3	1.1 (5)	C2—C3—C4—Cl2	179.87 (19)
Cl1—C2—C3—N2	−0.1 (4)	C2—C3—C4—C5	0.2 (4)
Cl1—C2—C3—C4	178.94 (19)	Cl2—C4—C5—N1	−179.2 (2)
C1—C2—C3—N2	−180.0 (3)	C3—C4—C5—N1	0.5 (5)

### 3,5-Dichloropyridin-4-amine Hydrogen-bond geometry (Å, º)

**Table:**

*D*—H···*A*	*D*—H	H···*A*	*D*···*A*	*D*—H···*A*
N2—H1*A*···Cl1	0.84 (4)	2.72 (3)	2.983 (2)	100 (3)
N2—H1*A*···Cl1^i^	0.84 (4)	2.81 (4)	3.625 (3)	164 (3)
N2—H2*A*···Cl2	0.85 (2)	2.66 (3)	3.020 (3)	107 (2)
N2—H2*A*···N1^ii^	0.85 (2)	2.16 (3)	2.931 (3)	149 (3)

Symmetry codes: (i) −*x*+1, −*y*+1, *z*+1/2; (ii) −*x*+1/2, *y*+1/2, *z*+1/2.
